# Levels of Anxiety and Fear among Nurses During the COVID-19 Pandemic: A Systematic Review

**DOI:** 10.1155/2023/2191984

**Published:** 2023-02-21

**Authors:** Cristina Morgado-Toscano, Juan Gómez-Salgado, Juan Jesús García-Iglesias, Javier Fagundo-Rivera, Daniel López-López, Regina Allande-Cussó

**Affiliations:** ^1^Health Sciences Doctorate School, University of Huelva, Huelva 21071, Spain; ^2^Health Sciences Research Unit: Nursing (UICISA: E), Nursing School of Coimbra (ESEnfC), Coimbra 3000, Portugal; ^3^Department of Sociology, Social Work and Public Health, Faculty of Labour Sciences, University of Huelva, Huelva 21007, Spain; ^4^Safety and Health Postgraduate Programme, Universidad Espíritu Santo, Guayaquil 092301, Ecuador; ^5^Centro Universitario de Enfermería Cruz Roja, University of Seville, Seville 41009, Spain; ^6^Health and Podiatry Group, Department of Health Sciences, Faculty of Nursing and Podiatry, Industrial Campus of Ferrol, Universidade da Coruña, Ferrol 15403, Spain; ^7^Department of Nursing, University of Seville, Seville 41009, Spain

## Abstract

**Aim:**

The aim of this review is to find out what levels of anxiety and fear have been shown by nurses during the COVID-19 pandemic.

**Background:**

Health security crises affect not only physical health but also the mental health and wellbeing of healthcare professionals due to a higher level of exposure. *Evaluation*. A systematic review was carried out following the PRISMA statement. Methodological quality was assessed using the Joanna Briggs Institute critical appraisal tools. The literature search was carried out in the PubMed, Scopus, and Web of Science (WoS) electronic databases based on the keywords that the research question yielded following the PECOT strategy. For the selection of articles, original articles, meta-analyses, systematic reviews, short communication articles, and case reports were included. Then, a series of inclusion and exclusion criteria were applied, screening the results to obtain a total of 18 articles, which were used to elaborate the study. *Key Issues*. Fear and anxiety levels were described in a total of 18 selected studies. The main fear-related concerns of the nurses were associated with the fear of infecting their family or friends and the fear of the death of a family member or friend.

**Conclusions:**

The main psychological impact on nurses during the COVID-19 pandemic was related to fear, anxiety, stress, and depression. Fear of infecting family members or of being infected were the main impacts perceived by nurses. *Implications for Nursing Management*. In general, high scores were found for levels of fear and anxiety, although the figures varied by country and time of data collection. Resilience was considered the main tool for coping with the loss and trauma experienced by nurses.

## 1. Background

On 31 December, 2019, the Wuhan Municipal Health and Sanitation Commission (Hubei, China) reported an outbreak in 27 people diagnosed with a type of pneumonia of unknown aetiology. All these people had in common that they had been in a seafood and poultry market in Wuhan in the previous days and this market was considered to be the epicentre of the outbreak [1].

The World Health Organization (WHO) has reported outbreaks caused by different infectious diseases over the past 20 years, such as severe acute respiratory syndrome (SARS) in 2003, influenza caused by the H1N1 subtype in 2009, Middle East respiratory syndrome in 2012, and Ebola virus in 2014 [2]. In late 2019, the Organization also reported on the presence of a new coronavirus (COVID-19) that was spreading rapidly worldwide [3], with the WHO declaring a pandemic on 11 March, 2020 [4].

This new virus has caused a drastic change in many aspects of our daily and working lives [5]. Today, it is impossible to estimate the number of people who have been infected. What is known is that the COVID-19 pandemic has put the healthcare system of many countries to the test. In response, many governments worldwide have implemented exceptional emergency measures, imposing severe confinement, quarantine, and social isolation measures [6]. Health security crises have been shown to generate stress and/or distress in the general population and even more so in healthcare workers [6].

Nurses have felt overwhelmed by heavy workloads during the COVID-19 pandemic, as they have been forced to prevent infections under adverse conditions, have close contact with confirmed cases, perform sample collection, and waste disposal [7].

At the organizational level, all care units have needed restructuring to cope with the disease (COVID-19 floors, adapted critical care units, clean pathway and COVID-19 pathway for the entry of patients, and relocation of staff) [8]. To this, the lack of material resources for patients should be added, such as respirators and nasal cannulas, and personal protective equipment for professionals such as gloves, gowns, and masks, which in some cases were reused [9], both in healthcare centres (hospitals and health centres) and in social-health centres (nursing homes). Both the lack of material and the close contact in the treatment of patients infected with SARS-CoV-2 place healthcare professionals at greater risk of infection, especially nurses, as they spend the most time with patients and perform techniques on patients the most [10].

In this pandemic context, many healthcare workers suffer from somatic symptoms such as cardiovascular, respiratory, neurological, and gastrointestinal symptoms, as well as psychological symptoms such as depression, fear, anxiety, and stress more frequently [11].

Fear and anxiety caused by possible illness can impose a high and destructive psychological burden, leading to mental problems, weakening of the immune system, and reduced body's ability to fight disease [12]. Anxiety is often related to feelings of unease and apprehension, being a psychological and physiological state characterised by cognitive, emotional, and behavioural factors [13].

Depending on the situation, these negative emotions can motivate actions, or on the contrary, contribute to an unwillingness to carry them out. Inhibiting the expression of these emotions leads to their intensification, or in the case of their maintenance, to emotional tension. According to experts, expressing negative emotions is beneficial and is recommended in psychotherapy [14].

Other determinants regarding physical and emotional health are sociodemographic characteristics. Demographic characteristics such as age, education, socioeconomic status, and nature of work are very important in explaining negative effects on psychological health [15]. These negative emotions, coupled with poor working conditions and limited resources, lead to a greater predisposition to incidents and failures in patient care, thus putting patient safety at risk [16].

The National Institute for Occupational Safety and Health (NIOSH) has ranked nursing as the 12^th^ most stressful profession and the most stressful of all healthcare professions [12]. In addition, the International Council of Nurses (ICN) reported that more than 600 nurses worldwide had died from COVID-19 as of 3 June, 2020. The first case of COVID-19 was confirmed in Ethiopia on 13 March, 2020, which was the first to be reported since the start of the outbreak in China in December, 2019. According to the National Ministry of Health, there were 31,336 COVID-19 cases and 544 (1.74%) deaths reported in Ethiopia as of 17 August, 2020 [1]. A study in China, where the COVID-19 outbreak began, assessed the psychological effects on 4692 nurses working in government-designated hospitals during the pandemic. The findings showed that the mental health of frontline nurses was generally poor, with 42.7% of them experiencing somatic symptoms, 9.4% depression, 8.1% anxiety, and 6.5% suicidal ideation [7].

The pandemic has affected not only physical health but also the mental health and wellbeing. The consequences can be particularly severe for healthcare professionals due to the higher level of exposure they experience. It is, therefore, crucial to estimate the psychological impact of the COVID-19 outbreak on healthcare workers, and more specifically on nurses because of their level of exposure [4]. The fact is that during the COVID-19 pandemic, nurses have been overwhelmed by heavy workloads, forced to prevent infections in adverse conditions, and having close contact with confirmed cases by taking samples or disposing of waste [7]. As a consequence, in this pandemic context, many healthcare workers have been suffering from somatic symptoms such as cardiovascular, respiratory, neurological, and gastrointestinal ones, as well as psychological symptoms such as depression, fear, anxiety, and stress more frequently than before [11].

Fear and anxiety derived from potential illness may impose a high and destructive psychological burden, leading to mental problems, weakening of the immune system, and reduced body's ability to fight disease [12]. Anxiety is often related to feelings of unease and apprehension, being both a psychological and physiological state characterised by cognitive, emotional, and behavioural factors [13].

Depending on the situation, these negative feelings can motivate actions, or on the contrary, contribute to an unwillingness to carry out certain protective behaviours. Inhibiting the expression of these emotions leads to their intensification, or if these are maintained, to emotional tension. According to experts, expressing negative emotions is beneficial and is recommended in psychotherapy [14].

Other determinants with regard to physical and emotional health are sociodemographic characteristics. Age, education, socioeconomic status, and nature of work are key in explaining negative effects on psychological health [15]. The same happens with sex. A report carried out by the European Parliament indicates that there are significant differences in mental health and wellbeing between men and women, highlighting in this report that women are more exposed to factors such as gender inequality, socioeconomic discrimination, overwork, gender-based violence, hunger, and malnutrition. These are all problems that significantly increase the likelihood of suffering from mental health problems among women. For all these reasons, it is estimated that broadly speaking, women show higher rates of depression, stress, anxiety, somatisation, and eating disorders than men [17].

In addition, in most societies, women are the main responsible ones for household chores (cooking, cleaning, and childcare), tasks that were accentuated during the confinement [18, 19]. Furthermore, according to the Official State Bulletin of Public Administrations, the percentage of employed women working in the Spanish health system is 74.2% [20].

What is certain is that these negative emotions, together with poor working conditions, and limited resources, lead to a greater predisposition to incidents and errors as regards patient care, which may in turn jeopardise patient safety [16].

Therefore, the aim of the present study was to identify the levels of anxiety and fear, as well as other associated symptoms, experienced by nurses during the COVID-19 pandemic. Secondary objectives are also defined such as the identification of the coping strategies used and the description of existing scales for the evaluation of the impact that the COVID-19 pandemic has had on mental health.

## 2. Methods

### 2.1. Study Design

Based on evidence-based medicine (EBM) [21] and following the criteria of the PRISMA statement [22], a systematic search for studies assessing the levels of fear and anxiety experienced by nurses during the COVID-19 pandemic was conducted. The followed protocol is listed in the International Prospective Register of Systematic Reviews (PROSPERO) with code CRD42022385193.

### 2.2. Search Strategy

The bibliographic search was carried out in the following electronic databases: PubMed, Scopus, and Web of Science (WoS), based on the keywords yielded by the research question that followed the PECOT strategy (Table 1). In addition, access was provided to websites of interest, such as those of the World Health Organization (WHO) and the National Statistics Institute (INE, for its Spanish acronym).

The descriptors used were as follows: nurse, COVID-19, anxiety, and fear. In order to enlarge the scope of the search, synonymous terms were used to complete the search based on the Medical Subject Headings (MeSH) thesaurus (Table 2), linked by the Boolean operators AND and OR.

Two researchers independently carried out the literature search and selected the articles included according to previously established criteria, subsequently agreeing on the results. Finally, Table 3 shows the search strategy used for each of the databases.

### 2.3. Selection Criteria

The inclusion criteria were the following: (1) written in English, Spanish, French, or Portuguese; (2) original articles, meta-analyses, systematic reviews, short communication articles, and case reports; (3) published from 2020 onwards; (4) population: nurses; and (5) articles assessing anxiety and fear levels, as well as other associated symptoms. Likewise, articles whose study population did not consist of nurses, or whose sample size was smaller than 50, as well as studies with low scientific-technical quality or whose sample consisted of students, were excluded from the sample.

### 2.4. Data Collection and Extraction

The final selection of the articles that compose the study sample, following the PRISMA 2020 guidelines for reporting systematic reviews [22], was peer-reviewed. All articles obtained as a result of the databases search were read by two different authors and screened and selected independently. Duplicate studies were removed, and the articles that could be included according to the previously established criteria, after reading the title and abstract, were selected. Then, two authors revised the text of the studies and decided to finally include or exclude them. A third author had the option to include a study or not in case of discrepancy. For the selection, data regarding authorship and year of publication, context, objective, type of study, participants, methods, main findings, and quality of the studies were compiled.

### 2.5. Methodological Quality Assessment

A critical appraisal tool for nonrandomised studies from the Joanna Briggs Institute (JBI) [23] of the University of Adelaide (Australia) was used to assess the methodological quality of the selected articles. There is a version for quantitative cross-sectional studies [24] (Supplementary Material 1) and for qualitative studies [25] (Supplementary Material 2) were used, with a cut-off point of 6 for inclusion in the review for quantitative studies and of 8 for qualitative studies.

## 3. Results

**Figure 1:**
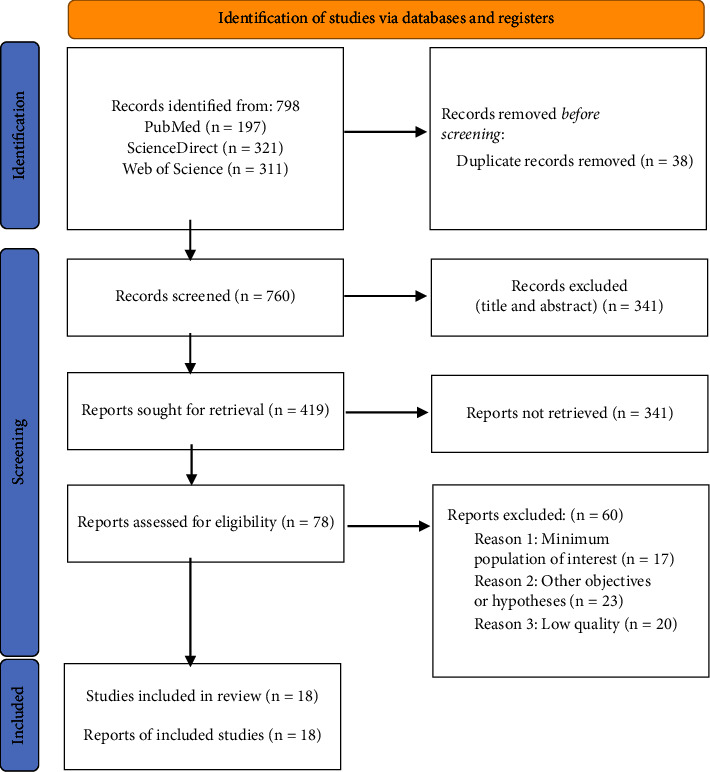
PRISMA flowchart.

The initial search identified a total of 798 references, which were then screened according to the topic of this review. A total of 18 studies were finally selected (Figure 1), 16 of them quantitative and 2 qualitative. Articles were found from different parts of the world, recruiting 4 from Spain, 2 from China, 3 from Saudi Arabia, 2 from Turkey, 1 from Ethiopia, 1 from Iran, 1 from Pakistan, 1 from Korea, 1 from Portugal, 1 from Jordan, and 1 from the United States.

In all the selected articles, the sample consisted of nurses; two of the articles also included nursing assistants. The included studies were assessed with the JBI critical appraisal tool, where they scored medium-high in both cross-sectional observational and qualitative studies.

Table 4, based on the Iberoamerican Cochrane Centre Handbook [33], shows the characteristics of each of the 18 studies included in this review. These were classified by author and year of publication, country, objective, design, participants, methods used, and main findings. In addition, the results of the JBI critical appraisal tool were added [34].

The main dimensions measured in the studies, in addition to anxiety and fear, were depression, stress, and others such as insomnia or certain coping strategies.

### 3.1. Fear

The only specific tool to measure fear in the selected studies was the Fear of COVID-19 Scale (FCV-19), designed by Ahorsu et al. in reference [35]. This has proven to be a valid and reliable instrument to specifically assess fear of COVID-19.

The study carried out by Simón Melchor et al. [6] in Spain, using the scale described previously, detected significant levels of fear in 46.9% of the sample, with a mean score for this scale of 21.54 points. Variables such as working in a COVID-19 unit, having more experience, being a specialised care nurse, and not living with relatives triggered higher levels of fear symptomatology [6].

The study conducted by Abid et al. [15] in Pakistan measured fear in the same way. In this case, the mean score was 23.92 points, confirming that high levels of fear of COVID-19 were strongly associated with negative emotional responses [15].

The mean score for this scale decreased in the study conducted by Cho and Kim [7]; in Korea, being 18.14. In terms of fear, statistically significant differences were found with respect to marital status, living with a partner, living with children, type of work related to COVID-19, and training in personal protection equipment (PPE) [7].

Finally, in the study conducted by Alnazly and Hjazeen [30] in Jordan, a mean score for the FCV-19 of 24.34 was obtained, indicating a remarkable fear of the COVID-19 pandemic. Female nurses were also found to have a higher mean score for fear of COVID-19 than their male counterparts [30].

The rest of the studies assessed fear using other types of questionnaires or surveys, and most of them agreed that the highest levels of fear were related to the fear of infecting their relatives or friends and the fear of death of one of them. This was stated in studies such as the one by Mekonen et al. [1], by Isik et al. [4], Altun Uğraş et al. [11], and more. This has been evidenced by some recorded expressions of nurses such as “I am petrified because when I go home, I am a danger to my family and a potential harm to them” [31]. Thus, the degree of exposure was positively associated with fear [5]. Fear and anxiety can also lead to such a destructive psychological burden that it may weaken the immune system, reducing the body's ability to fight disease [12].

### 3.2. Anxiety, Depression, and Stress

Many of the selected articles measured anxiety, stress, and depression together using the 21-item depression, anxiety, and stress scale (DASS-21) [36]. Through this scale, these articles revealed that the prevalence of anxiety, depression, and stress among nurses was 69.6%, 55.3%, and 20.5%, respectively, with a 95% CI. Also, the likelihood of anxiety was three times higher among nurses who did not have a COVID-19 management guide and four times higher in those with a chronic disease [1].

In another study, according to the DASS scale, 46.4% of the sample showed moderate to extremely severe anxiety, and those nurses who had had or were suffering from COVID-19 and who had a risk comorbidity were found to be at higher risk of anxiety. In the case of depression, 66.7% reported moderate to severe depression. Nurses who had had or were suffering from COVID-19 infection, with work experience of more than 10 years, aged 46 years or older, and with risk comorbidity due to COVID-19 were at a higher risk of depression. Finally, 18.2% of the sample had moderate stress. Those who were suffering or had suffered from COVID-19 infection and who were working more hours per month than the normal working day were at a higher risk of experiencing stress [6].

In the study by Abid et al. [15], the same scale was used to detect that 66% of the sample suffered from anxiety, 55% from depression, and 49% from stress.

Another study using the DASS scale found that 78.0% of the sample suffered from anxiety, of whom 16.6% reported symptoms of mild anxiety, 19.5% of moderate anxiety, 14.6% of severe anxiety, and 27.2% of most severe anxiety. 84.3% showed signs of depression, with 11.7% being mild, 36.9% moderate, 19.2% severe, and 16.3% most severe. Finally, 65.74% showed signs of stress, with 16.3% showing mild, 23.4% moderate, 20% severe, and 6.1% most severe symptoms [4].

Likewise, the study carried out by Sampaio et al. [27] reported that anxiety symptoms decreased over time. Possible predictors of changes in anxiety scores were sex, age, nursing speciality, number and quality of face masks, number, and quality of PPE. They concluded that the greater the fear, the more anxiety symptoms. With regard to depression, male participants had a lower mean score for depression compared to females. As with anxiety, male nurse specialists were found to have a lower score for depression, and here the rule that the higher the fear, the more symptoms of depression was also true. For stress, the mean score remained almost stable over time. Men, with experience, and specialists had lower scores for stress. The higher the fear, the more stress symptoms [27].

Finally, the study by Alnazly and Hjazeen [30] also used the DASS scale, concluding that 73.8% of the sample had an extremely severe level of anxiety, 43.8% had a moderate level of depression, and 45.4% had a severe level of stress [30].

Anxiety and depression were also measured together using the Hospital Anxiety and Depression Scale (HADS). This scale assesses the severity of symptoms and occurrence of anxiety and depressive disorders in somatic, psychiatric, and primary care patients and also in the general population [37].

The data showed that in the first period, 68.3% of the subjects suffered from anxiety, and 18.2% were at risk of it. The results also showed that during the first period, 49.6% of the subjects were depressed, and 21.4% were at risk of depression. All scores decreased in the second period compared to the first [28].

One of the instruments used to specifically measure anxiety was the Templer questionnaire, which in this case, specifically measures the level of death anxiety [38]. This identified levels of mild and severe anxiety in 30.9% and 69.1% of the nurses, respectively [12].

Another instrument used was the Generalised Anxiety Disorder Scale (GAD-7), developed by Spitzer et al. [39], with the percentage range of nurses suffering from anxiety being between 30% and 40% and from severe anxiety ranging from 1.2% to 2.4%. In this case, the mean number of daily working hours was significantly associated with anxiety [13]. This same scale was also used by Cho and Kim [7], and a mean score for this scale of 5.01 was detected, indicating mild anxiety symptoms. In this study, anxiety was affected by work experience of more than 3 months and time in charge of work related to COVID-19.

Anxiety was also specifically measured through the Self-Rating Anxiety Scale (SAS) compiled by Zung [40]. Among the participants, 34% reported mild anxiety levels, 3.53% moderate anxiety, and 0.44% severe anxiety. Being a woman, having less rest time, having children, lacking confidence in fighting the pandemic, regretting being a nurse, and fear of contagion in the family were risk factors for reporting anxiety [26].

The other authors opted to use their own questionnaires to measure anxiety. This was the case of Muñoz–Muñoz et al. [5], where it was concluded that female nurses were more likely to suffer from anxiety (59.3%), compared to men (43.4%). Similarly, Altun Uğraş et al. [11] stated that 73.4% of the sample had anxiety related to the lack of healing treatment for the disease and 71.8% related to a possible second and third wave of the pandemic. The study conducted by Tayyib and Alsolami [3] indicated high levels of anxiety (7.76 out of 10). Using the same method, Alsharif [29] confirmed that 20% of the sample rated their level of anxiety about the infection as 10 out of 10. The overall mean for anxiety levels was 5.7 on the 10-point Likert scale.

In the case of depression, to specifically measure this construct, Cho and Kim [7] used the Patient Health Questionnaire created by Kroenke et al. [41]. In this questionnaire, Cronbach's alpha was 0.86 for patients with COVID-19, which confirmed its relationship with depression [7].

In the case of stress, some authors also used specific scales such as the Perceived Stress Scale (PSS) developed by Cohen et al. [42]. Through this scale, 41.4% of the participants reported stress. This study showed that a stable and safe work environment is a key factor in reducing perceived stress [13]. This same scale was used by Cui et al. [26], finding excessive stress in 32.23%. Fear of infecting family members, the regret of being a nurse, and the number of night shifts in a week were positively correlated with reported stress [26]. Other authors used their own questionnaires to assess stress, as was the case of Muñoz–Muñoz et al. [5]; in which 52.2% considered that they had felt much more psychological stress than usual. In the study by Natividad et al. [25], the highest scores for stress were identified in nurses who believed they could transmit COVID-19 to their family and friends, followed by the inadequacy of the PPE and that they could be positive for the disease whenever they had respiratory symptoms.

Overall, in the study by Huerta-González et al. [32], the main fears expressed by nurses were infecting family members or being infected, followed by the fear of death of patients. In the case of Guttormson et al. [31], participants frequently shared concern for family and friends. Some nurses even put their decision to become a nurse to question and reported overwhelming distress suffered on a daily basis. Some nurses also reported a lack of understanding of their environment.

### 3.3. Other Symptoms

Another assessed symptom was insomnia. This was carried out through specific tools such as the Insomnia Severity Index (ISI) [43]. It was possible to detect insomnia in 77.6% of the sample. Of these, 55.9% suffered from moderate to severe insomnia. Nurses who were suffering or had suffered from COVID-19, who were working more hours per month than the normal working day, and who did so in specialised care had a higher risk of suffering from insomnia. On the contrary, those with more leisure activities had a lower risk [6]. Another scale to measure sleep quality used by another study was the Athens Insomnia Scale (AIS) [44], detecting insomnia in 41.5% of the sample [13]. Using their own questionnaires, the study carried out by Sampaio et al. [27] detected a large number of nurses with poor sleep quality, which decreased significantly over time.

The presence of emotional exhaustion and personal fulfilment was also assessed using the Maslach Burnout Inventory (MBI) [45]. Nurses who had suffered or were suffering from COVID-19, with work experience of more than 10 years, who were working more hours per month than the normal working day, and whose workstation was in a COVID-19 unit were at greater risk of presenting high emotional exhaustion. On the other hand, nurses who had suffered or were suffering from COVID-19, who worked shifts, who had comorbidity risk for COVID-19, and who worked in specialised care were more at risk of presenting a low level of personal fulfilment [6].

Finally, in one of the studies, with the aim of specifically measuring coping strategies, the Coping Style or Simplified Coping Style Scale (SCSQ) [46] was used, which concluded that negative coping behaviours such as fantasy, avoidance, self-blame, and indulgence have a negative impact on psychological well-being [26]. In this line, another study concluded that resilience, understood as the ability to adapt flexibly to changes caused by stressful events and to recover from negative emotional experiences, was considered by nurses as the main coping technique to counteract the losses and traumas experienced [32].

## 4. Discussion

The aim of this review was to assess the fear and anxiety experienced by nurses during the COVID-19 pandemic. To do so, levels of fear and anxiety were analysed in a total of 18 selected studies, where data on other mental health problems such as depression, stress, and insomnia were also provided.

The main findings of this systematic review are based on the fact that the only tool found that assesses any mental health problem specific to the COVID-19 pandemic is the Fear of COVID-19 Scale (FCV-19), created by Ahorsu et al. [35]. This scale was used by several selected studies, with scores generally higher than 20 in almost all studies, which indicated high levels of fear of the COVID-19 pandemic. The main fear complaints nurses expressed were related to fear of infecting their family or friends and fear of the death of a family member or friend.

No specific scale to measure COVID-19 anxiety was found in the selected articles, with most authors opting for the Depression, Anxiety, and Stress Scale (DASS) [36]. In general, high scores were obtained for these three constructs, although the figures varied from study to study, considering that they were developed in different countries and at different times.

However, it is known that there is a specific scale to measure fear and anxiety in the face of COVID-19. In Spain, a group of researchers designed the so-called COVID-19 Anxiety and Fear Assessment Scale (AMICO). It is composed of 16 items and proved to be a reliable and valid tool to be used as a screening instrument [47]. Probably, one of the reasons why this scale has not been used in the selected articles is because, so far, it has only been validated in Spain and is in the process of being validated in other countries such as the United Kingdom and Portugal.

Differences may have also been influenced by the timing of the study and the different restrictive measures in place at the time in each country, which varied widely throughout the pandemic. For example, a study by Burton et al. [48] reported that rates of suicidal ideation had increased during the first weeks of confinement in countries such as the UK. Similarly, a study by Gasteiger et al. [49] found that depression and anxiety had been most negatively affected during the first ten weeks of social distancing during the COVID-19 pandemic. All these negative emotions can cause problems at the psychological level and, of course, in work carried out by the nursing staff, bringing about errors, and malpractice, which would also influence patient safety [50, 51].

A cross-sectional study revealed that nurses working on the frontline during the COVID-19 pandemic, i.e., in ICU and emergency departments, reported workload and compassion fatigue, with associated mental health consequences [52]. However, other studies showed that frontline nurses may have shown less concern and fear for their safety, as they believed in the effectiveness of their protective equipment, whereas many clinical nurses, not on the frontline, who encountered problems with ineffective and insufficient medical protection, became anxious and worried, especially at the beginning of the pandemic, when there was a lack of medical equipment [53]. Therefore, it can be said that risk perception is a mental psychological construct that is subject to cognitive, emotional, socio-cultural, political, and personal variables [54]. It would therefore be necessary to assess more specific groups within society.

In addition to these, some risk factors potentially associated with mental health symptoms among nurses have also been identified, which can be divided into four major sections [27]:

1. Personal factors, such as age, sex, and nursing specialisation
2. Working conditions, such as the existence of adequate PPE.
3. Family dynamics, such as being far from home.
4. Attitude towards COVID-19, such as fear of getting infected or fear of infecting others.

Among the limitations of the current study, it is worth mentioning that all articles written in languages other than Spanish, Portuguese, or English were rejected; this may have led to excluding articles that met the rest of the inclusion requirements. Also, studies conducted in China were included, and, being the country where the COVID-19 outbreak started, the sample might have different characteristics from others when assessing potential mental health-related problems. It should also be noted that the incidence of cases worldwide was not uniform, nor were the proposed restrictions. Another possible limitation could be that the studies found were conducted on different dates, which may have affected the results due to the progression of the pandemic itself. Likewise, there has been variability in the instruments used by the different authors to measure the same construct, which may also be a limitation of this study.

Regarding the applicability of this study, it is worth highlighting the need to identify the impact that the COVID-19 pandemic has had on nurses in different cultural contexts, as a starting point, based on a systematic and rigorous review, for the design and implementation of support or follow-up strategies by managers. This study also highlights the need to study the impact that the COVID-19 pandemic has had on other groups of health and nonhealth professionals because all of them make up the work team that provides patient care and attention and are therefore susceptible to receiving help and support from the health managers of each health centre.

## 5. Conclusion

The results of the study show that the main psychological impacts on the nurses during the COVID-19 pandemic were fear, anxiety, stress, and depression and, as a consequence of these, other problems such as insomnia. Fear of infecting family members or being infected and fear of the death of a loved one were the main impacts perceived by the nurses.

Resilience was considered the main tool to cope with the losses and traumas experienced by the nurses.

The impact that the COVID-19 pandemic has had on physical and mental health identified in this review should provide the basis for new studies to identify the prevalence of the main symptoms described, following the chronological and epidemiological evolution of the pandemic, as well as the study of their relationship with different levels of care or areas of specific clinical care. This could generate new strategies to improve nurses' coping strategies in health crisis situations, as well as encourage leadership and management procedures to be put in place by managers during these situations. The finding obtained in the present study also allows for the possibility of creating tools to assess the impact that these crisis situations have at all professional levels as a measure of prevention and screening of occupational health problems that could indirectly affect the quality of the services provided.

## 6. Implications for Nursing Management

Although the vast majority of the referred studies affirm a high prevalence of psychological distress among healthcare professionals who have been active during COVID-19 [55], there are some differences with respect to the results found in the different studies included in this systematic review. This may be due to the fact that, in some selected studies, the sample of nurses worked specifically in the ICU, a unit with a high care burden during the COVID-19 outbreak; other nurses worked exclusively with COVID-positive patients; and in other studies, the area of work was not specified, but they were focused on the general nursing population. In this regard, a study focusing on healthcare professionals by Li et al. [56] concluded that frontline workers experienced greater mental health deterioration during the pandemic than other healthcare workers who were not so exposed.

## Figures and Tables

**Table 1:** PECOT format: keywords.

Population	Nurses
Effect	Fear and anxiety level
Comparator	Other variables
Outcomes	Prevalence of anxiety-fear, predisposing/protective factors, differences between countries and services, frontline vs. nonfrontline differences, and differences/fluctuations over time
Time	During the COVID-19 pandemic
Research question	What levels of anxiety and fear have nurses shown during the COVID-19 pandemic?

**Table 2:** Search terms.

MeSH	Terms
Nurse	Healthcare professionals OR healthcare workers OR healthcare providers OR nurse
COVID-19	COVID-19
Anxiety	Anxiety
Fear	Fear

**Table 3:** Search strategy in each database.

Database	Search strategy	Search date	Outcomes	Selected
PubMed	(nurs^*∗*^ AND (COVID-19 AND (anxiety AND fear))	03/08/22	197	18
WoS	nurs^*∗*^ (topic) AND fear AND anxiety (topic) AND COVID-19 (topic)	03/08/22	311	34
Scopus	((nurs^*∗*^) AND (anxiety AND fear) AND (COVID-19))	03/08/22	321	26
Total			798	78

**Table 4:** Characteristics of the studies included in the systematic review.

Studies	Context	Objective	Type of study	Participants	Methods	Main findings	JBI
Reference [1]	North-west Amhara (Ethiopia)	To assess the prevalence and associated factors of anxiety, depression, and stress in nurses	Cross-sectional quantitative study	995 nurses in Amhara hospitals	DASS-21	The prevalence of anxiety, depression, and stress was 69.6%, 55.3%, and 20.5%, respectively. Some factors that were significantly associated with anxiety, depression, or stress were workload, sex, having adequate protective equipment, having children, living with people over 60 years old, having a chronic illness, and having a history of mental disorders, among others.	7/8

Reference [12]	Tehran (Iran)	To assess the relationship between COVID-19 and death anxiety in nurses	Cross-sectional quantitative study	110 nurses working in the ICU in Tehran's hospitals	Templer scale	Mild anxiety levels were identified in 30.9% of the sample, and severe anxiety in 69.1%. The results also showed that the level of death anxiety was associated with age, working hours per week, having children, exposure to patient death, and satisfaction with personal protective equipment.	6/8

Reference [6]	Huesca (Spain)	To analyse the psycho-emotional impact of COVID-19 on nurses in the province of Huesca	Cross-sectional quantitative study	179 nurses working during the pandemic in Huesca	(i) DASS-21 (ii) FCV-19S (iii) ISI (iv) MBI	Depression was diagnosed in 16.8% of the sample, anxiety in 46.4%, and stress in 22.4%. Insomnia was detected in 77.6%, and a high score for burnout syndrome in 50.0%. The FCV-19S scale detected fear in 46.9% of the sample. The data revealed that variables such as spending more time at work, having less leisure time, a greater work experience, and the presence of risk comorbidities for COVID-19, among others, constitute precipitating factors for suffering from mental health problems.	7/8

Reference [5]	Spain	To analyse the psychosocial impact of the COVID-19 pandemic on ICU nurses in Spain	Cross-sectional quantitative study	456 ICU nurses who had worked during the COVID-19 pandemic in Spain	Self-administered questionnaire created ad hoc	53.1% had experienced difficulty concentrating at work; for 63.8%, work performance had been negatively affected; 81.8% reported feeling much more tired; 89.9% had problems sleeping; 53.5% felt much more anxious than usual; 82.5% of participants felt down, depressed, or hopeless; 52.2% had felt much more psychological stress than usual; 56.8% felt much more fearful than usual; 18.2% had increased their use of psychotropic drugs. Finally, women were more prone to anxiety than men.	7/8

Reference [13]	Wuhan (China)	To investigate anxiety levels in nurses and the association with perceived stress and insomnia	Cross-sectional quantitative study	643 nurses working on the frontline with COVID-19 patients from 3 to 10 March 2020 in hospitals in China	(i) Socio-demographic questionnaire (ii) GAD-7 (iii) Questionnaire regarding the COVID-19 environment (iv) AIS	33.4% of the nurses surveyed reported episodes of anxiety which they associated with factors such as perceived stress and insomnia. Significant associations were detected between anxiety, perceived stress, insomnia, working four night shifts per week, work experience, and fear of COVID-19.	7/8

Reference [15]	Gujrat (Pakistan)	To determine the predictive association between fear of COVID-19 and emotional distress in nurses	Cross-sectional quantitative study	Nursing staff in public and private sector hospitals in Gujrat (*n* = 500)	(i) FCV-19S (ii) DASS-21	71% were women. The mean score for the FCV-19 was 23.92. For the DASS-21, it was 32.15. Fear of COVID-19 was a significant predictor of stress, depression, and anxiety in nurses. A significant mediating role of demographic variables such as sex, age, education, and marital status was detected in the predictive association between fear of COVID-19 and emotional distress in nurses.	6/8

Reference [4]	Turkey	To determine the levels of depression, anxiety, and stress in the mental health of nurses during COVID-19	Cross-sectional quantitative study	Nurses actively working in the public and private sectors in Turkey (826)	DASS-21	84.3% showed symptoms of depression, 78% anxiety, and 65.74% stress. The most important problems included equipment shortages, administrative problems, and issues such as accommodation and food, and these had a statistically significant correlation with the nurses' levels of depression, anxiety, and stress. Taking the necessary steps to address problems and fears is important to protect the health, productivity, and efficiency of nurses during the pandemic period.	6/8

Reference [7]	Korea	To identify factors affecting fear, anxiety, and depression in nurses working on the frontline with COVID-19 patients	Cross-sectional quantitative study	975 nurses who had contact with COVID-19 patients for 3 months	(i) Hospital safety climate survey (ii) FCV-19 (iii) GAD-7 (iv) PHQ	COVID-19 increased nurses' levels of fear, anxiety, and depressive symptoms. Hospital safety climate influenced nurses' mental well-being. In terms of fear, statistically significant differences were found with regard to marital status, living with a partner, living with children, and type of work. There were significant differences in the level of anxiety according to living together, type of work, and work experience longer than 3 months. Depressive symptoms varied with age, educational level, and annual income.	

Reference [25]	Saudi Arabia	To assess nurses' feelings towards COVID-19 and determine stressors while exploring coping strategies and coping factors	Cross-sectional quantitative study	Nurses working in the Ministry of Health hospitals (Saudi Arabia) caring for patients with COVID-19 (*n* = 313)	(i) MERS-CoV staff questionnaire (ii) Brief COPE	Women, married, with a bachelor's degree, and aged 25–34 years had more significant coping strategies. Most were nervous about the risk of contracting the disease, unhappy about working overtime, and frightened about exposure to the virus. Factors that increased stress were the thought that they could transmit the virus, the lack of PPE, and that they could be positive when they had symptoms. They found comfort in their religion, advice from friends, and family. Women were more able to use coping strategies.	6/8

Reference [11]	Turkey	To examine health problems and stressors related to patient care in ICU nurses during COVID-19	Cross-sectional quantitative study	Nurses working in the ICU and caring for adult patients with COVID-19 in Turkey (*n* = 1140)	Self-administered questionnaire created ad hoc	The majority of the sample was female, with a mean age of 29.7 years. Of the total, only 15.6% experienced health problems during the pandemic. The most common psychological complaints were anxiety (3%), insomnia (1.1%), and depression (0.4%). 92.3% were afraid of carrying COVID-19 and infecting their loved ones, 78.9% were afraid of being separated from loved ones, 74.4% were afraid of getting sick with COVID-19, 73.4% were anxious about the lack of curative treatment for the disease, and 71.8% were anxious about the possible second and third waves of the pandemic.	6/8

Reference [26]	Jiangsu province (China)	To identify the impact of COVID-19 on the psychology of Chinese nurses in emergency departments and fever clinics and to identify associated factors	Cross-sectional quantitative study	Nurses working in hospitals in the province of Jiangsu and who were exposed to COVID-19 for more than one month (*n* = 437)	(i) Self-rating anxiety scale (ii) Perceived stress scale-14 (iii) Simplified coping style questionnaire	96.47% were women, with a mean age of 33.15 years. 34% reported mild anxiety, 3.53% moderate, and 0.44% severe. Excessive stress was detected in 32.23%. Being a woman, having less time off, having children, lack of confidence, regrets about being a nurse, and fear of contagion were risk factors for anxiety. Participants with positive attitudes, who did not regret being a nurse, who were trained in emergencies, who were willing to go to Hubei province to be rescued, and who did not fear infecting others responded more positively to stress.	6/8

Reference [27]	Portugal	To assess variations in sleep quality and symptoms of depression, anxiety, and stress among nurses during the COVID-19 outbreak, as well as to assess risk factors	Prospective cohort study	Frontline nurses in Portugal during the COVID-19 pandemic (*n* = 829)	DASS-21	81.4% were women with a mean age of 39.0 years. In the DASS-21, there was a tendency for a decrease in all three scores over time. Predictors of change in depression were sex, age, nursing specialty, number and quality of face masks, quality of gowns, quality of glasses, fear of infection, and infection. As regards stress, predictors of change were sex, age, nursing specialty, number of gloves, quality of face masks, quality of gowns, quality of glasses, fear of infection, and infection. A large number of nurses had poor sleep quality, decreasing over time.	7/8

Reference [28]	Spain	To determine symptoms of depression and/or anxiety among nurses and nursing assistants during the periods known as the first and second waves of COVID-19	Cross-sectional quantitative study	Practising nurses or auxiliary nurses in Spain during the first and second waves of COVID-19 (*n* = 627)	HADS	At baseline, 28.4% reported sleep disturbances every day. In the first period, 68.3% suffered from anxiety, and 18.2% were at risk of it. All scores decreased in the second period. During the first period, 49.6% were depressed, and 21.4% were at risk of depression. All scores decreased in the second period compared to the first one. The results showed that in the first period, the percentages of anxiety and depression were lower in the nurses' group than in the auxiliary nurses' group. In the second period, the frequencies decreased in both.	7/8

Reference [3]	Saudi Arabia (KSA)	To assess the psychological effects derived from fear and stress levels due to COVID-19 on KSA nurses	Cross-sectional quantitative study	Nurses working in hospitals during the COVID-19 outbreak and who understand English (*n* = 300)	Self-administered questionnaire created ad hoc	The majority were women. Nurses had high levels of anxiety and stress during the COVID-19 outbreak. Participants reported that their work put them at high risk of infection, caused them a lot of stress at work, and they had a high level of fear of transmitting COVID-19 to their families, friends, and colleagues. The results pointed to some predictors that increased the nurses' level of fear, such as social networks, exposure to pre-outbreak trauma, and availability to care for infected patients.	7/8

Reference [29]	Saudi Arabia (KSA)	To assess nurses' knowledge of COVID-19 and level of anxiety about the COVID-19 outbreak in the current pandemic situation	Cross-sectional quantitative study	87 nurses from the medical and surgical departments at King Abdul Aziz University Hospital in Jeddah working with COVID-19 patients	Self-administered questionnaire created ad hoc	A minority (35.6%) rated their level of anxiety about infecting their loved ones as 10 out of 10, although the mean was 5.7 on the Likert scale. Respondents had adequate and good knowledge about the causes, transmission, symptoms, treatment, and mortality rates of COVID-19 (71.90%). The main sources of information for nurses were social networks (51.7%) and the World Health Organization, and the Ministry of Health (36.8%).	6/8

Reference [30]	Amman (Jordan)	To assess levels of psychological distress among nurses during COVID-19 and to determine associated factors and coping strategies	Cross-sectional quantitative study	Nurses from hospitals located in the Amman area and who have provided care to patients with COVID-19 or suspected COVID-19 (*n* = 130)	(i) FCV-19S (ii) DASS (iii) Brief coping inventory	Nurses have moderate levels of fear and depression. Anxiety and fear were positively correlated. Female nurses had greater psychological distress and fear than male nurses. Nurses who cared for COVID-19 positive patients and those who had a friend or family member who had tested positive had higher levels of fear and psychological distress. Working longer hours was moderately related to fear and anxiety. Nurses were also found to generally adopt maladaptive coping styles.	6/8

Reference [31]	United States	To describe the experiences of ICU nurses during the COVID-19 pandemic in the United States	Cross-sectional quantitative study	Nurses working in ICUs in the USA during the COVID-19 pandemic (*n* = 285)	Electronic survey	Nurses working in the ICU in the US during the COVID-19 pandemic experienced an increased burden due to lack of treatment, poor prognosis, and lack of family presence. The physical, emotional, and psychological impact resulted in burnout, anxiety, insomnia, and distress. The pandemic led some nurses to question their decision to become nurses. Compared to other healthcare workers, nurses reported higher levels of anxiety and depression. Despite the challenges, they identified positive aspects.	

Reference [32]		To analyse and synthesise qualitative studies investigating nurses' perceptions of the psychological impacts of COVID-19	Systematic review of qualitative studies	Qualitative studies that analysed nurses' perceptions of the psychological impacts of COVID-19	Qualitative systematic review	The main psychological impacts perceived by frontline nurses were fear, anxiety, stress, social isolation, depressive symptoms, uncertainty, and frustration. Fear of infecting or being infected was the main perceived impact. They experienced loneliness and a high level of anxiety, higher than that of the general population. Nurses felt that resilience helped to counteract the losses and trauma they experienced.	

## Data Availability

All data generated or analysed during this study are included in this published article (and its supplementary information files).

## Abbreviations

AIS: Athens Insomnia Scale
AMICO: COVID-19 Anxiety And Fear Assessment Scale
CI: Confidence interval
COVID-19: Coronavirus disease 2019
DASS-21: 21-item Depression, Anxiety, and Stress Scale
EBM: Evidence-based medicine
FCV-19: Fear of COVID-19 scale
GAD-7: Generalised Anxiety Disorder Scale
HADS: Hospital Anxiety and Depression Scale
ICN: International Council of Nurses
ICU: Intensive care unit
ISI: Insomnia Severity Index
JBI: Joanna Briggs Institute
MBI: Maslach burnout inventory
MeSH: Medical subject headings
NIOSH: National Institute for Occupational Safety and Health
PECOT: Population, effect, comparison, outcomes, time, research question
PPE: Personal protection equipment
PRISMA: Preferred reporting items for systematic reviews and meta-analyses
PSS: Perceived Stress Scale
SARS: Severe acute respiratory syndrome
SAS: Self-rating Anxiety Scale
SCSQ: Coping style or Simplified Coping Style Scale
WHO: World Health Organization
WoS: Web of science.

## Supplementary Materials

Supplementary Materials A critical appraisal tool for nonrandomised studies from the Joanna Briggs Institute (JBI) [23] of the University of Adelaide (Australia) was used to assess the methodological quality of the selected articles. There is a version for quantitative cross-sectional studies [24] (Supplementary Material 1) and for qualitative studies [25] (Supplementary Material 2) were used, with a cut-off point of 6 for inclusion in the review for quantitative studies and of 8 for qualitative studies.
