# Supplementary material for: Levels of Anxiety and Fear among Nurses During the COVID-19 Pandemic: A Systematic Review
Source: J Nurs Manag. 2023 Feb 21;2023:2191984. doi: 10.1155/2023/2191984 (PMC11918982; doi:10.1155/2023/2191984)
Supplement: Supplementary Materials — A critical appraisal tool for nonrandomised studies from the Joanna Briggs Institute (JBI) [23] of the University of Adelaide (Australia) was used to assess the methodological quality of the selected articles. There is a version for quantitative cross-sectional studies [24] (Supplementary Material 1) and for qualitative studies [25] (Supplementary Material 2) were used, with a cut-off point of 6 for inclusion in the review for quantitative studies and of 8 for qualitative studies. [file 2191984.f1.docx]

*SUPPLEMENTARY MATERIAL 1*

*Scores of cross-sectional observational studies*

| Study | JBI | The participants and the environment are described in detail | Inclusion criteria are clearly defined | Exposure was validly and reliably measured | The criterion used to measure the condition was objective | Confounding factors were identified | Strategies for dealing with confounding factors | Valid and reliable measured results | Appropriate statistical analysis was used |
| --- | --- | --- | --- | --- | --- | --- | --- | --- | --- |
| Mekonen et al., 2020 | 7/8 | ☺ | ☺ | ☺ | ☺ | ☹ | ☺ | ☺ | ☺ |
| Belash et al., 2021 | 6/8 | ☺ | ☺ | ☺ | ☺ | ☹ | ☹ | ☺ | ☺ |
| Melchor et al., 2022 | 7/8 | ☺ | ☺ | ☺ | ☺ | ☺ | ☹ | ☺ | ☺ |
| Muñoz-Muñoz et al., 2022 | 7/8 | ☺ | ☺ | ☺ | ☺ | ☺ | ☹ | ☺ | ☺ |
| Shen et al., 2021 | 7/8 | ☺ | ☺ | ☺ | ☺ | ☺ | ☹ | ☺ | ☺ |
| Abid et al., 2021 | 6/8 | ☺ | ☺ | ☺ | ☺ | ☹ | ☹ | ☺ | ☺ |
| Işık et al., 2022 | 6/8 | ☺ | ☺ | ☺ | ☺ | ☹ | ☹ | ☺ | ☺ |
| Cho et al., 2021 | 6/8 | ☺ | ☺ | ☺ | ☺ | ☹ | ☹ | ☺ | ☺ |
| Natividad et al., 2021 | 6/8 | ☺ | ☺ | ☺ | ☺ | ☹ | ☹ | ☺ | ☺ |
| Altun Uğraş et al., 2022 | 6/8 | ☺ | ☺ | ☺ | ☺ | ☹ | ☹ | ☺ | ☺ |
| Cui et al., 2021 | 6/8 | ☺ | ☺ | ☺ | ☺ | ☹ | ☹ | ☺ | ☺ |
| Sampaio et al., 2021 | 7/8 | ☺ | ☹ | ☺ | ☺ | ☺ | ☺ | ☺ | ☺ |
| Sánchez-Sánchez et al., 2021 | 7/8 | ☺ | ☺ | ☺ | ☺ | ☺ | ☹ | ☺ | ☺ |
| Tayyib & Alsolami, 2020 | 7/8 | ☺ | ☺ | ☺ | ☺ | ☺ | ☹ | ☺ | ☺ |
| Alsharif et al., 2021 | 6/8 | ☺ | ☺ | ☺ | ☺ | ☹ | ☹ | ☺ | ☺ |
| Alnazly et al., 2021 | 6/8 | ☺ | ☺ | ☺ | ☺ | ☹ | ☹ | ☺ | ☺ |

Yes: ☺; No: ☹; Unclear or not applicable: 😐

*SUPPLEMENTARY MATERIAL 2*

*Scores of qualitative studies*

| Study | JBI | Congruence between stated philosophical perspective and research methodology | Congruence between methodology and question/objectives | Consistency between the methodology and the method used to collect data | Consistency between methodology and data representation and analysis | Consistency between methodology and interpretation of results | Cultural and theoretical localisation | Influence of the researcher on the sample and vice versa | Representativeness of participants | Ethical approval by an appropriate body | Relationship between findings and data analysis or data interpretation |
| --- | --- | --- | --- | --- | --- | --- | --- | --- | --- | --- | --- |
| Guttormson et al., 2022 | 8/10 | ☺ | ☺ | ☺ | ☺ | ☺ | ☺ | 😐 | 😐 | ☺ | ☺ |
| Huerta-González et al., 2021 | 8/10 | ☺ | ☺ | ☺ | ☺ | ☺ | 😐 | 😐 | ☺ | ☺ | ☺ |

Yes: ☺; No: ☹; Unclear or not applicable: 😐
